# Supplementary material for: Association of Novel Advanced Glycation End-Product (AGE10) with Complications of Diabetes as Measured by Enzyme-Linked Immunosorbent Assay
Source: J Clin Med. 2021 Sep 29;10(19):4499. doi: 10.3390/jcm10194499 (PMC8509253; doi:10.3390/jcm10194499)
Supplement: Supplementary file 1 [file jcm-10-04499-s001.zip › jcm-1387713-supplementary/jcm-1387713-supplementary.pdf]

Table S1. Conditions of the chromatographic separation of samples 1(A), 1(B_0), 1(B_1) and 1(B_2)

Time [min]	Flow (µL/min)	% A (water)	% B (acetonitrile)
1	0.3	97	3
10	0.3	97	3
11	0.3	60	40
15	0.5	5	95
20	0.5	97	3

Table S2. Summary of spectrometric analysis conditions for the tested compound (mass standards)

Parameter	Value
Voltage applied to the capillary	3.0 [kV]
Voltage applied to the cone	40 [kV]
Temperature of the source	80 °C
Cone gas flow	52 - 25 L/h
Atomizing gas pressure	0.15 Ba
Purge gas	200 h
